# Remnant cholesterol associates with hypertension beyond low-density lipoprotein cholesterol among the general US adult population

**DOI:** 10.3389/fendo.2023.1260764

**Published:** 2023-09-29

**Authors:** Liu Shi, Dongmei Zhang, Jianqing Ju, Anlu Wang, Tianyi Du, Xuanye Chen, Yewen Song, Zhuye Gao, Hao Xu

**Affiliations:** ^1^ Graduate School, Beijing University of Chinese Medicine, Beijing, China; ^2^ National Clinical Research Center for Chinese Medicine Cardiology, Xiyuan Hospital, China Academy of Chinese Medical Sciences, Beijing, China; ^3^ Dongzhimen Hospital, Beijing University of Chinese Medicine, Beijing, China

**Keywords:** remnant cholesterol, hypertension, low-density lipoprotein cholesterol, apolipoprotein B, dyslipidemia

## Abstract

**Background:**

Previous findings have indicated that elevated low-density lipoprotein cholesterol (LDL-C) and remnant cholesterol (RC) are associated with hypertension. We aim to explore whether higher RC levels may be associated with hypertension beyond LDL-C in the general US adult population.

**Methods:**

This study included 10,842 adults from the National Health and Nutrition Examination Survey (NHANES) 1999–2018. Weighted multivariable logistic regression models were used to estimate the odds ratios (ORs) of hypertension for LDL-C and RC. We also performed analyses examining the association between hypertension and LDL-C vs. RC concordant/discordant groups.

**Results:**

A total of 4,963 (41.54%, weighted) individuals had hypertension. The weighted median levels were LDL-C: 118mg/dL, RC: 20mg/dL. At lower LDL-C clinical cut-point, the proportion of discordantly high RC dramatically increased. After multivariable adjustment, log RC was associated with higher prevalence of hypertension [OR 2.54, 95% confidence interval (CI) 2.17–2.99]. Participants with the highest tertile of RC were more likely to have hypertension (OR 2.18; 95% CI 1.89-2.52) compared with those with the lowest tertile of RC. This association remained marked after including body mass index (BMI), LDL-C, high-density lipoprotein cholesterol (HDL-C) or triglycerides. The association between LDL-C and hypertension was absent after adjusting for BMI, RC or triglycerides. Compared with low LDL-C/low RC group, the discordant low LDL-C/high RC group was associated with hypertension (OR 2.04; 95% CI 1.72-2.42), whereas the high LDL-C/low RC group was not, regardless of BMI, HDL-C or triglycerides. Similar results were observed when examining discordance among different clinical cut-points, except for the cut-point of LDL-C 70 mg/dL and RC 13 mg/dL. To better understand the association, we performed an additional analysis, which showed that among participants with apolipoprotein B < median (92mg/dL), those with discordant RC ≥ median (20mg/dL) had significantly higher odds of having hypertension (OR 1.73; 95% CI 1.38-2.17).

**Conclusion:**

RC was associated with hypertension beyond LDL-C in the general US adult population. This association went beyond increased triglycerides levels, and lipoproteins other than apoB may be involved.

## Introduction

Hypertension is one of the most critical risk factors for heart disease and stroke, two of the leading causes of premature death in the United States (1). Nearly 47.3% of US adults have hypertension (2). The total costs of hypertension as a risk factor could increase to 30% of the total expenditure for cardiovascular disease, by 2035 (3).

Hypertension and hypercholesterolemia are important and prevalent disorders in atherosclerotic cardiovascular disease (ASCVD). More than half of hypertensive patients have hypercholesterolemia (4). Abnormal lipid metabolism induces insulin resistance, chronic inflammatory response, and impaired endothelial function, all of which may affect blood pressure (5). Triglyceride-rich lipoproteins (TRLs) and their cholesterol-rich remnants are now recognized as powerful causative factors of atherosclerosis and metabolic disorders, with predictive ability exceeding that of low-density lipoprotein cholesterol (LDL-C) (6–8). Remnant cholesterol (RC) is the cholesterol content of TRLs and its metabolic remnants. Previous studies have shown that both LDL-C and RC were associated with the onset of hypertension (9–11). A Chinese cohort-based study revealed that RC was associated with the risk of atherosclerosis and atherosclerotic advancement, even in individuals with favorable LDL-C levels (12). Another community-based cohort indicated that there was a threshold effect of LDL-C on the new-onset hypertension, and the risk was increased only at normal LDL-C concentrations (13).

These unanticipated observations have spurred calls to validate these findings further in other populations settings and to re-assess the independent pathogenic role of RC. In the present study, we intended to examine the association of RC and LDL-C with hypertension in the general US adult population with not receiving lipid-lowering treatment, and to further identify the concordant/discordant LDL-C and RC associations with hypertension.

## Methods

### Study setting and population

We used data collected in the National Health and Nutrition Examination Survey (NHANES), which is conducted by the National Center for Health Statistics (NCHS) of the Center for Disease Control and Prevention. NHANES is a nationally representative survey of the civilian, noninstitutionalized US population, to evaluate the health and nutritional status. All participants provided written informed consent. The NHANES study protocols were approved by the NCHS ethics review board. Data were collected consecutively but released in 2-year cycles (14). Specific information is available at the NHANES website (https://www.cdc.gov/nchs/nhanes/index.htm). We examined data from 10 sequential cycles covering the periods 1999-2018. There were 17,398 participants aged 18 years or older with plasma lipid data available as well as complete questionnaire information and physical examination data. We excluded participants who were pregnant (N=567). Considering that some medications for cancer may affect blood pressure, we also excluded participants who had cancer (N=2,071). Next, we excluded those did not self-report medication use (N=19) or currently on lipid-lowering treatment (N=3,429) that may affect plasma lipid levels. Participants with missing key laboratory data (N=36) were also excluded. Considering the complex, multi-stage, probability sampling design of NHANES, we further excluded individuals with missing weights (N=434). The final analytical cohort included 10,842 participants.

### Hypertension diagnosis

Blood pressure measurements were taken by the mobile examination center examiners using a standardized protocol. Hypertension was diagnosed based on the questionnaires and physical examination results. Three or 4 sometimes consecutive blood pressure determinations were obtained. Traditional averages did not represent the final blood pressure results. If only one blood pressure measurement was available, that measurement is the average. If there were multiple blood pressure readings, the first reading was always not included in the average. Participants were regarded as having hypertension if they met one of the following 3 criteria were considered to have hypertension: (1) the average systolic blood pressure ≥130 mmHg or the average diastolic blood pressure ≥80 mmHg; (2) the answer to the question “are you now taking prescription for hypertension” was “yes”; (3) the answer to the question “have you ever been told that you had high blood pressure” was “yes”.

### Lipid measurements

Fasting total cholesterol (TC) and triglycerides (TG) were analyzed using enzymatic methods. High-density lipoprotein cholesterol (HDL-C) was determined by the heparin manganese precipitation or direct immunoassay method. LDL-C was calculated using the **Martin-Hopkins equation** as it employs an adjustable factor, the ratio of TG to very low-density lipoprotein cholesterol (VLDL-C), to estimate LDL-C levels (15). Considering the greater inaccuracy of LDL-C estimation at higher TG levels, especially TG > 400 mg/dL, the use of estimating equations in this situation is not currently recommended (16). Therefore, we estimated LDL-C only for participants with TG ≤ 400 mg/dL. RC (mg/dL) was estimated as TC (mg/dL) minus calculated LDL-C (mg/dL) minus HDL-C (mg/dL). LDL-C and RC were also estimated using the Friedewald equation and Sampson-NIH (National Institute of Health) formula, which have been presented in the published paper (17). Apolipoprotein B (apoB) was measured by immuno-turbidimetric assay for 5 consecutive cycles covering the periods 2007-2016.

### Concordance/discordance definition

Since there is no recognized physiologically discordant cut-point between different lipid measures, we adopted different thresholds to identify the LDL-C and RC concordant/discordant groups. First, we used the medians as cut-points. The population was divided into 4 groups: (1) low LDL-C/low RC (LDL-C < median, RC < median); (2) low LDL-C/high RC (LDL-C < median, RC > median); (3) high LDL-C/low RC (LDL-C > median, RC < median); (4) high LDL-C/high RC (LDL-C > median, RC > median). Second, we also focused on relevant clinical LDL-C cut-points (70, 100, and 130 mg/dL) according to established guideline recommendations (5, 18, 19). The equivalent population percentile corresponding to these LDL-C values was used to determine the respective RC cut-points.

### Covariates

Information about participants age, sex, race/ethnicity, family income-poverty ratio, education level, smoking status, disease status and use of antihypertensive and hypoglycemic medications was collected from household interviews using questionnaires. Body mass index (BMI), waist circumference (WC) and alcohol intake were obtained when participants took physical examinations. Additionally, considering the interconnection between the metabolic abnormalities, we also included glycosylated hemoglobin A1c (HbA1c), fasting blood glucose (FBG), estimated glomerular filtration rate (eGFR), and high-sensitivity C-reactive protein (hs-CRP) in the analysis. Description of laboratory methodology can be found in the laboratory procedures manual for the corresponding cycle. The specific definitions and classifications of covariates were shown in the **Supplemental Material**.

### Statistical analysis

NHANES uses a complex, multi-stage, probability sampling design. Consistent with NHANES analysis recommendations (20), we newly constructed weights for combining survey cycles. New weights were calculated as two-tenths of WTSAF4YR (a fasting weight variable in NHANES) for 1999-2000 and 2001-2002 survey cycles, one-tenths of WTSAF2YR for 2003-2004, 2005-2006, 2007-2008, 2009-2010, 2011-2012, 2013-2014, 2015-2016, 2017-2018 waves.

Characteristics were described depending on whether participants had hypertension, RC levels and LDL-C and RC concordant/discordant groups. Data were presented as mean ± standard error for continuous variables or frequency (weighted percentage) for categorical variables. We used weighted linear regression model (continuous variables) or weighted chi-square test (categorical variables) to compare baseline characteristics. Weighted multivariable logistic regression models adjusting for potential confounders were used to estimate odds ratios (ORs) of hypertension for LDL-C and RC. LDL-C and RC were included in the model as continuous variables (log-transformed) and categorical variables (tertiles), respectively. In the multivariate models, we adjusted for age (continuous), sex (male or female), race/ethnicity (non-Hispanic white, non-Hispanic black, Mexican American, or other), educational level (less than high school, high school or equivalent, or college or above), family income-poverty ratio (≤ 1.0, 1.1-3.0, or > 3.0), smoking status (never smoker, former smoker, or current smoker), and alcohol drinking (non-drinker, low to moderate drinker, or heavy drinker) in model 1. Model 2 further adjusted for chronic kidney disease (yes or no), diabetes mellitus (yes or no), coronary heart disease (yes or no) in addition to covariates in model 1. In model 3, we further adjusted for eGFR (continuous), FBG (continuous), and HbA1c (continuous). To analyze the association of RC with hypertension independent of LDL-C, we also further adjusted for LDL-C in model 4. In our main analyses, we did not adjust for BMI and WC as they could be mediators on the pathway from lipid disorders to hypertension. The median value was assigned to each tertile as a continuous variable to test for a linear trend. The association of LDL-C and RC concordant/discordant groups with hypertension was also evaluated adjusting for variables as described above. Considering that the nonlinear relationship may affect the rationality of discordant/concordant grouping, we examined LDL-C and RC as nonlinear variables, modeled with 3-knotted restricted cubic spline regression, adjusting all variables in model 3 mentioned above. Before performing regression analyses, we first assessed the collinearity of RC/LDL-C and adjusted covariates. Using variance inflation factor as an assessment tool, covariates with variance inflation factor above 10 were considered as collinear variables, and those with variance inflation factor between 5 and 10 could be excluded as needed. Collinearity test results showed no high collinearity between RC/LDL-C and adjusted covariates (**Supplemental Tables S1-S4**).

Stratified analyses and interaction analyses were conducted to examine whether the association differed by age quintiles, sex (male or female), obesity status (BMI < 25, 25 ≤ BMI < 30, or BMI ≥ 30 kg/m^2^), smoking status (never smoker, former smoker, or current smoker), chronic kidney disease (yes or no), diabetes mellitus (yes or no), survey cycles (1999-2008 or 2009-2018) and hypoglycemic drugs use (yes or no). The *P* values for the product terms between LDL-C and RC discordant/concordant groups and stratification variables were used to evaluate the significance of interactions.

Additional sensitivity analyses were performed to access the robustness of the findings. First, we further adjusted for BMI and WC in addition to model 3, given the possibility that they could be confounders (i.e., affecting lipid levels) rather than mediators (i.e., affected by lipid levels). Collinearity screening results showed that the variance inflation factors for BMI and WC ranged from 5 to 10, so we only further adjusted for BMI (**Supplemental Tables S5-S7**). Second, considering that differences in HDL-C levels between LDL-C and RC discordant/concordant groups may affect the association results, we further adjusted for HDL-C in addition to model 3. Third, as the levels of TG and RC were correlated, TG was further adjusted for based on model 3. Fourth, to examine whether inflammation had an effect on the observed correlation, we further adjusted for hs-CRP in a subgroup of the study participants (only available in NHANES 2015-2018, N=1,947). We appropriately excluded variables with variance inflation factor greater than 10 to ensure the reliability of the regression results (**Supplemental Tables S8-S10**). Fifth, LDL-C and RC were recalculated using the Friedewald equation and Sampson-NIH formula to determine the effect of different calculation methods. Sixth, considering the effect of recall bias, we re-conducted the analysis after excluding people who were previously told they had hypertension. Seventh, to rule out a possible effect of antihypertensive drugs on lipids, we performed additional analyses in the population not taking antihypertensive drugs. Finally, given that apoB reflects more accurately the number of atherogenic lipoprotein particles in the blood than LDL-C (21), we explored the association between apoB and RC discordant/concordant subgroups (the medians as cut-points) and hypertension (apoB data only available in NHANES 2007-2016, N=6,854). Data were analyzed from November 2022 to May 2023. All statistical analyses were conducted using R software version 4.2.2 (R foundation for Statistical Computing, Vienna, Austria). All *p* values were two-sided with a significance level of < 0.05.

## Results

Of 10,842 participants, 5,048 (weighted proportion, 48.17%) were men; the mean (standard error) age was 42.2 (0.21) years; 41.54% (weighted) individuals had hypertension. The weighted median levels were LDL-C: 118mg/dL, RC: 20mg/dL, apoB: 92mg/dL. **Supplemental Table S11 and S12** described the characteristics of the participants according to hypertension status and tertiles of RC levels, which were unevenly distributed among them. A greater number of hypertensive participants were in low LDL-C/high RC group and high LDL-C/high RC group. Compared with individuals in high LDL-C/low RC group, those in low LDL-C/high RC group tended to be younger, women, Mexican American, current smokers and heavy drinkers; were less likely to have higher education levels and family income; had higher BMI, WC, TG and hs-CRP levels but lower HDL-C, non-HDL-C, TC and apoB levels; had higher prevalence of comorbidities and higher rates of antihypertensive and hypoglycemic drugs use. (**Table 1**).

**Table 1:** Characteristics of participants in different concordant/discordant groups ^*^.

Characteristic	Concordant/discordant of LDL-C and RC				*P* value
	Low LDL-C and low RC (n=3,517)	Low LDL-C and high RC (n=1,853)	High LDL-C and low RC (n=1,558)	High LDL-C and high RC (n=3,914)	
Age, (mean ± SE), years	37.70 ± 0.30	40.75 ± 0.41	44.79 ± 0.44	46.12 ± 0.29	<0.001
Sex					
Male	1951 (57.22)	754 (40.59)	763 (50.47)	1580 (42.23)	<0.001
Female	1566 (42.78)	1099 (59.41)	795 (49.53)	2334 (57.77)	
Race/ethnicity					
Non-Hispanic White	1517 (67.09)	833 (67.48)	670 (67.75)	1889 (72.90)	<0.001
Non-Hispanic Black	888 (13.95)	280 (8.55)	440 (15.73)	495 (6.65)	
Mexican American	519 (7.59)	419 (11.12)	205 (6.01)	922 (9.74)	
Other	593 (11.37)	321 (12.85)	243 (10.50)	608 (10.71)	
Educational level					
Less than high school	209 (3.08)	177 (4.52)	115 (3.65)	484 (5.68)	<0.001
High school or equivalent	1214 (30.12)	707 (34.73)	545 (32.05)	1541 (38.23)	
College or above	2094 (66.80)	969 (60.75)	898 (64.30)	1889 (56.09)	
Family income-poverty ratio					
≤1.0	719 (14.27)	385 (14.47)	256 (9.90)	723 (12.59)	<0.001
1.1-3.0	1383 (34.68)	789 (37.87)	598 (33.79)	1650 (36.02)	
>3.0	1415 (51.05)	679 (47.66)	704 (56.32)	1541 (51.38)	
Smoking status					
Never smoker	1985 (55.81)	872 (48.17)	890 (56.29)	1834 (47.10)	<0.001
Former smoker	714 (21.55)	454 (24.15)	360 (22.71)	1065 (26.32)	
Current smoker	818 (22.64)	527 (27.67)	308 (21.00)	1015 (26.57)	
Alcohol drinking					
Non-drinker	478 (10.61)	348 (14.55)	260 (14.52)	773 (16.46)	<0.001
Low to moderate drinker	1544 (45.44)	590 (33.37)	680 (46.32)	1381 (38.24)	
Heavy drinker	1495 (43.94)	915 (52.08)	618 (39.16)	1760 (45.30)	
BMI	26.29 ± 0.17	29.70 ± 0.21	27.76 ± 0.19	29.82 ± 0.16	<0.001
Waist Circumference	90.43 ± 0.42	100.88 ± 0.52	95.01 ± 0.46	101.70 ± 0.36	<0.001
SBP, mmHg	115.63 ± 0.33	121.23 ± 0.44	118.68 ± 0.44	122.57 ± 0.33	<0.001
DBP, mmHg	68.21 ± 0.26	71.76 ± 0.35	70.83 ± 0.32	73.31 ± 0.24	<0.001
Hypertension	1190 (28.84)	954 (48.72)	680 (38.16)	2139(51.55)	<0.001
Diabetes mellitus	238 (4.27)	274 (11.02)	113 (4.88)	516(9.55)	<0.001
Coronary heart disease	29 (0.45)	37 (1.21)	12 (0.52)	49(0.82)	0.007
Chronic kidney disease	314 (6.95)	277 (11.51)	136 (6.62)	531(10.25)	<0.001
Stroke	41 (0.79)	35 (1.12)	26 (0.86)	82(1.70)	0.002
Antihypertensive drugs use	135 (2.86)	156 (7.23)	84 (4.43)	307 (6.87)	<0.001
Hypoglycemic drugs use	100 (1.63)	135 (5.84)	43 (1.75)	176 (3.01)	<0.001
LDL-C, (mean ± SE), mg/dL	89.34 ± 0.48	99.75 ± 0.41	139.12 ± 0.64	150.85 ± 0.54	<0.001
HDL-C, (mean ± SE), mg/dL	60.32 ± 0.42	47.78 ± 0.41	60.12 ± 0.46	48.28 ± 0.28	<0.001
Non-HDL-C, (mean ± SE), mg/dL	104.40 ± 0.51	126.26 ± 0.47	155.86 ± 0.65	180.64 ± 0.61	<0.001
TG, (mean ± SE), mg/dL	66.45 ± 0.41	160.95 ± 1.71	71.56 ± 0.44	163.94 ± 1.26	<0.001
TC, (mean ± SE), mg/dL	164.72 ± 0.59	174.04 ± 0.56	215.97 ± 0.81	228.93 ± 0.68	<0.001
RC, (mean ± SE), mg/dL	15.06 ± 0.06	26.51 ± 0.19	16.74 ± 0.06	29.79 ± 0.17	<0.001
FBG, (mean ± SE), mmol/L	5.35 ± 0.02	5.76 ± 0.05	5.47 ± 0.03	5.79 ± 0.03	<0.001
HbA1c, (mean ± SE), %	5.26 ± 0.01	5.45 ± 0.03	5.40 ± 0.02	5.55 ± 0.02	<0.001
eGFR, (mean ± SE), mL/min/1.73 m^2^	103.51 ± 0.48	100.25 ± 0.57	97.21 ± 0.53	94.86 ± 0.39	<0.001
ApoB, mg/dL ^†^	71.32 ± 0.43	83.83 ± 0.48	101.60 ± 0.68	117.00 ± 0.64	<0.001
Hs-CRP, mg/L ^‡^	3.37 ± 0.33	4.09 ± 0.35	3.37 ± 0.36	3.92 ± 0.33	0.273

apoB, apolipoprotein B; BMI, body mass index; DBP, diastolic blood pressure; eGFR, estimated glomerular filtration rate; FBG, fasting blood glucose; HbA1c, glycosylated hemoglobin A1c; hs-CRP, high-sensitivity C-reactive protein; HDL-C, high-density lipoprotein cholesterol; LDL-C, low-density lipoprotein cholesterol; RC, remnant cholesterol; SBP, systolic blood pressure; TG, triglycerides; TC, total cholesterol.

SI conversions: to convert LDL-C, HDL-C, Non-HDL-C, TC, and RC to mmol/L, multiply by 0.02586; to convert TG to mmol/L, multiply by 0.01129.

^*^ The different concordant/discordant groups based on LDL-C 118 mg/dL and RC 20 mg/dL cut-points. All means and SEs for continuous variables and percentages for categorical variables were weighted, with the exception of the number of participants.

^†^ ApoB data only available in 2007-2016.

^‡^ hs-CRP data only available in 2015-2018.

### Distribution of LDL-C and RC

**Figure 1:**
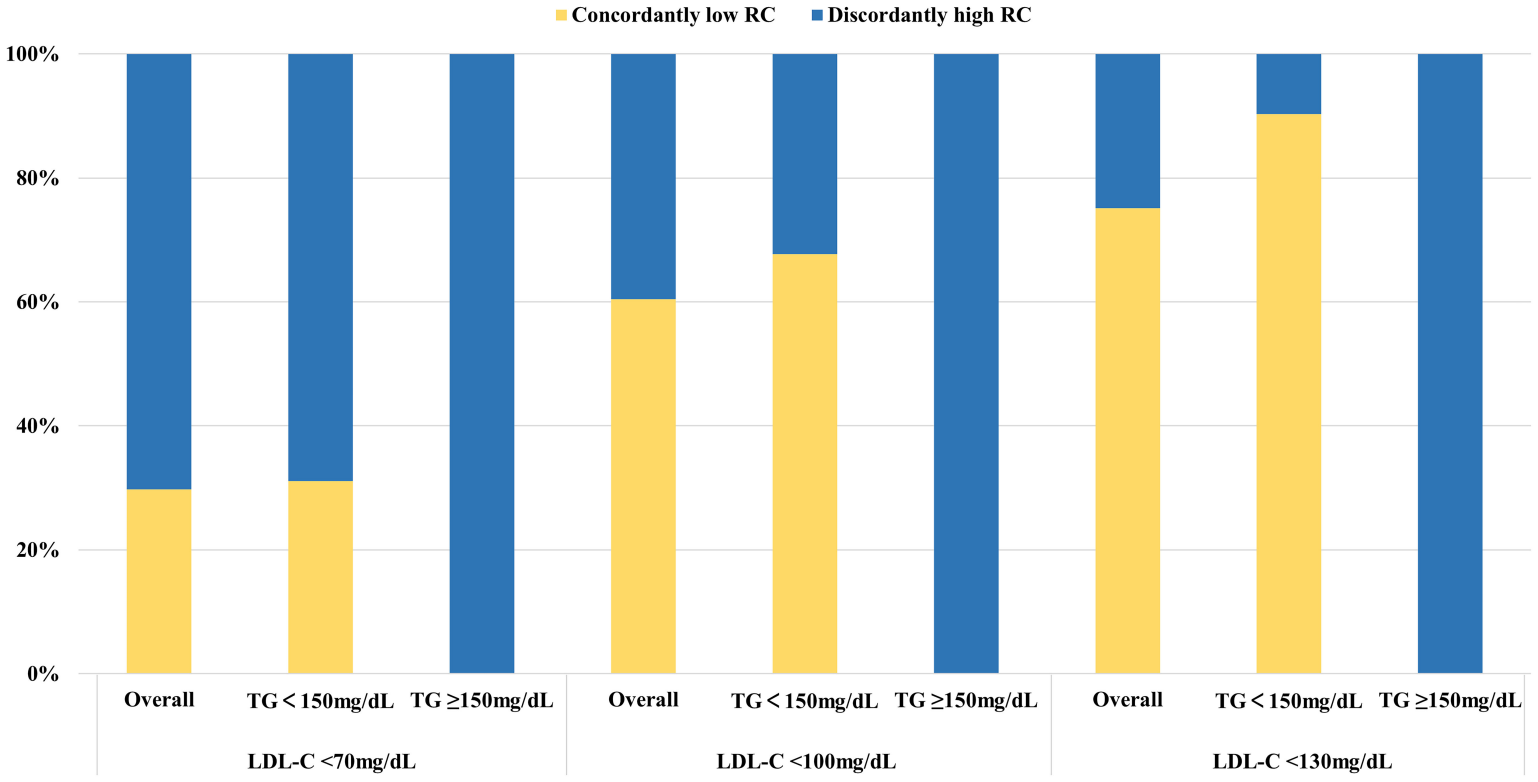
Proportions of concordance/discordance among individuals with LDL-C below clinical cut-points in the NHANES 1999 to 2018. Discordantly high RC definition: LDL-C < 70mg/dl, RC > 13mg/dl; LDL-C < 100mg/dl, RC > 17mg/dl; LDL-C < 130mg/dl, RC > 23mg/dl. LDL-C cut-points (70, 100, and 130 mg/dL) were based on established guideline recommendations. The equivalent population percentile corresponding to these LDL-C values was used to determine the respective RC cut-points. Percentages were weighted. LDL-C, low-density lipoprotein cholesterol; RC, remnant cholesterol; TG, triglycerides.

High levels of RC accounted for a relatively large proportion of the participants with increased LDL-C levels (**Supplemental Figure S1**). All participants with LDL-C below the clinical cut-points had discordantly high levels of RC when TG ≥150mg/dL. At lower LDL-C clinical cut-point, the proportion of discordantly high RC dramatically increased, up to 70.26% (weighted) in those with LDL-C < 70mg/dL (**Figure 1**).

### Association between LDL-C or RC with hypertension

**Figure 2:**
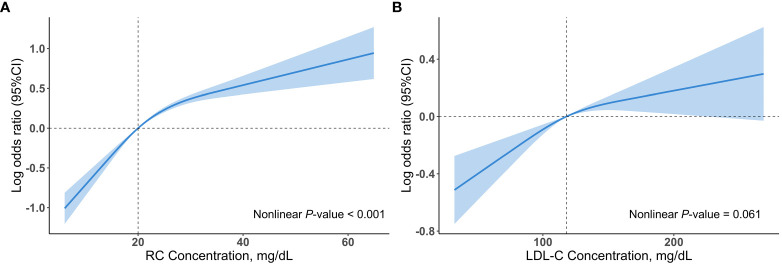
Associations (log odds ratios, 95%CIs) of RC and LDL-C concentrations with hypertension using a restricted cubic spline regression model in the NHANES 1999 to 2018. **(A)** Association between RC concentrations and hypertension. **(B)** Association between LDL-C concentrations and hypertension. Results were adjusted for age (continuous), sex (male/female), race/ethnicity (non-Hispanic white, non-Hispanic black, Mexican American, other), educational level (less than high school, high school or equivalent, college or above), family income-poverty ratio (≤1.0, 1.1-3.0, >3.0), smoking status (never smoker, former smoker, current smoker), alcohol drinking (non-drinker, low to moderate drinker, heavy drinker), chronic kidney disease (yes or no), diabetes mellitus (yes or no), coronary heart disease (yes or no), eGFR (continuous), FBG (continuous), and HbA1c (continuous). All estimates accounted for complex survey design. Restricted cubic spline regression model was conducted with 3 knots. Shadows represent the 95% CIs for the spline model (with respective medians as reference). CI, confidence interval; LDL-C, low-density lipoprotein cholesterol; RC, remnant cholesterol.

Multivariable restricted cubic spline regression models showed a monotonically increasing relationship between RC or LDL-C and hypertension, even though the nonlinear *p*-value for RC < 0.001 (**Figure 2**). After adjusting for several potential confounders, we observed a significant association between log RC levels with hypertension (OR, 2.54; 95% CI, 2.17–2.99) in model 3, with unchanged results after adjusting for LDL-C (OR, 2.69; 95% CI, 2.26–3.21) (**Table 2**). The results were similar when RC was included in the model as categorical variables (tertiles) (**Table 2**). This association remained marked after including BMI or HDL-C (model 1 and model 2, **Supplemental Table S13**). Notably, tertiles of RC continued to be associated with hypertension despite adjusting for TG (model 3, **Supplemental Table S13**). In contrast, the association between tertiles of LDL-C and hypertension was absent after adjusting for BMI, RC or TG (**Table 2** and **Supplemental Table S13**).

**Table 2:** Adjusted ORs (95%CIs) of hypertension according to LDL-C and RC concentrations.

Variables	LDL-C and RC Levels, OR (95%CI)				Per 1 log-transformed increment, mg/dL
	Tertile1	Tertile2	Tertile3	*P*_trend_	
LDL-C					
Median (range), mg/dL	86.8 (19.6-104.4)	118.4 (104.5-133.6)	153.1 (133.7-354.1)		
Cases, No./Total No.	1,343/3,613	1,671/3,615	1,949/3,614		4,963/10,842
Model 1 ^*^	Ref	1.20 (1.05-1.37)	1.28 (1.11-1.48)	0.001	1.52 (1.26-1.85)
Model 2 ^†^	Ref	1.23 (1.08-1.40)	1.33 (1.14-1.54)	<0.001	1.58 (1.29-1.93)
Model 3 ^‡^	Ref	1.22 (1.07-1.40)	1.32 (1.13-1.54)	<0.001	1.57 (1.27-1.94)
Model 4 ^§^	Ref	0.99 (0.86-1.14)	0.89 (0.75-1.07)	0.186	0.86 (0.69-1.09)
RC					
Median (range), mg/dL	14.6 (2.9-17.4)	20.5 (17.5-24.5)	30.7 (24.6-67.5)		
Cases, No./Total No.	1,238/3,615	1,667/3,602	2,508/3,625		4,963/10,842
Model 1 ^*^	Ref	1.49 (1.31-1.69)	2.33 (2.02-2.68)	<0.001	2.74 (2.34-3.20)
Model 2 ^†^	Ref	1.47 (1.28-1.67)	2.22 (1.93-2.56)	<0.001	2.59 (2.21-3.03)
Model 3 ^‡^	Ref	1.45 (1.27-1.65)	2.18 (1.89-2.52)	<0.001	2.54 (2.17-2.99)
Model 4 ^||^	Ref	1.46 (1.27-1.67)	2.21 (1.89-2.58)	<0.001	2.69 (2.26-3.21)

CI, confidence interval; LDL-C, low-density lipoprotein cholesterol; OR, odds ratio; RC, remnant cholesterol.

^*^ Model1: adjusted for age (continuous), sex (male/female), race/ethnicity (non-Hispanic white, non-Hispanic black, Mexican American, other), educational level (less than high school, high school or equivalent, college or above), family income-poverty ratio (≤1.0, 1.1-3.0, >3.0), smoking status (never smoker, former smoker, current smoker), and alcohol drinking (non-drinker, low to moderate drinker, heavy drinker).

^†^ Model 2: further adjusted (from Model 1) for chronic kidney disease (yes or no), and diabetes mellitus (yes or no), coronary heart disease (yes or no).

^‡^ Model 3: further adjusted (from Model 2) for eGFR (continuous), FBG (continuous), and HbA1c (continuous).

^§^ Model 4: further adjusted (from Model 3) for log RC (continuous).

^||^ Model 4: further adjusted (from Model 3) for log LDL-C (continuous).

All estimates accounted for complex survey design.

### Association between LDL-C and RC concordant/discordant groups with hypertension

**Figure 3:**
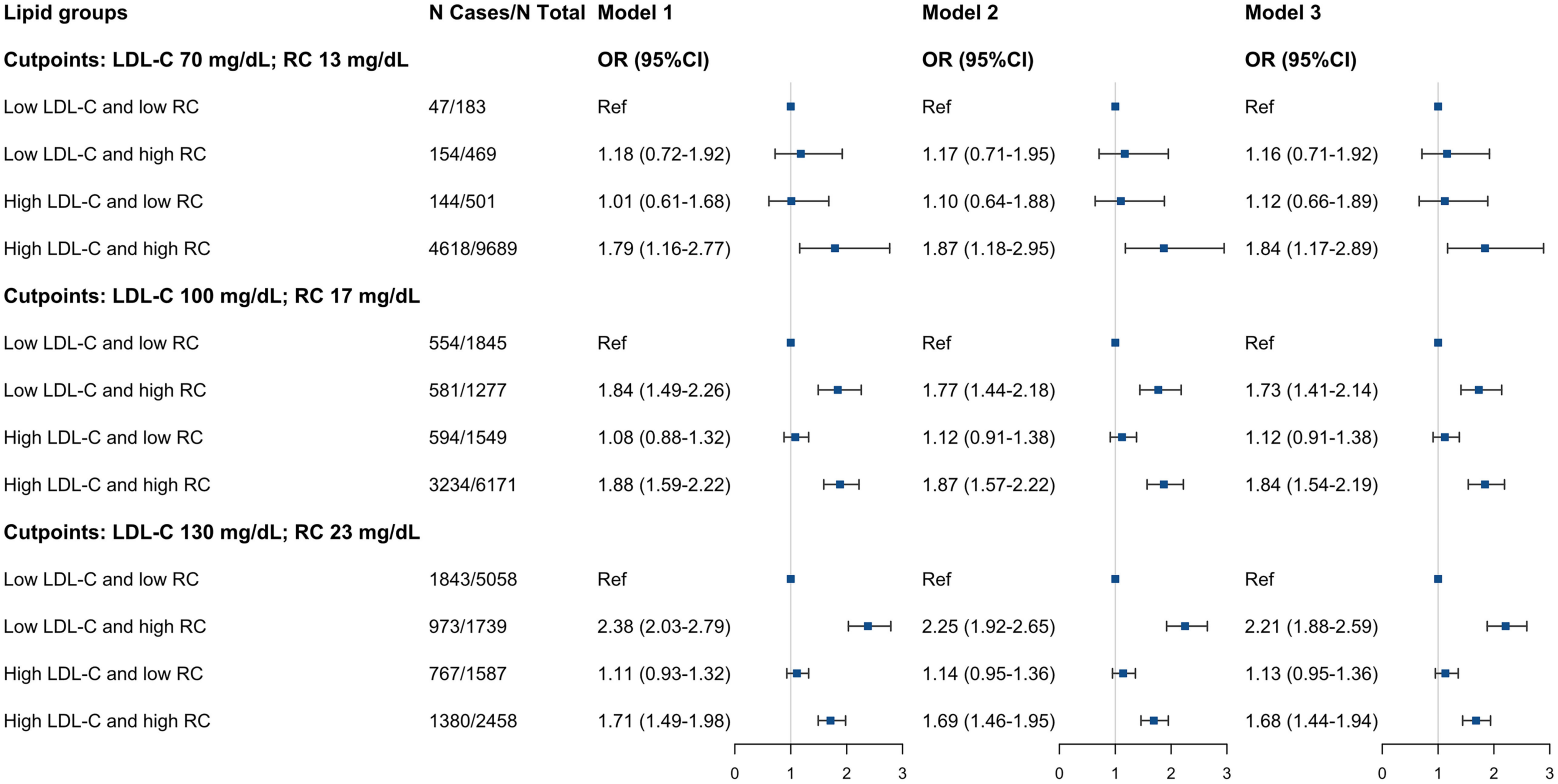
Adjusted ORs (95%CIs) of hypertension according to different concordant/discordant groups by LDL-C clinical cut-points (70, 100, and 130 mg/dL) and percentile-equivalents for RC in the NHANES 1999 to 2018. Model 1: adjusted for age (continuous), sex (male/female), race/ethnicity (non-Hispanic white, non-Hispanic black, Mexican American, other), educational level (less than high school, high school or equivalent, college or above), family income-poverty ratio (≤1.0, 1.1-3.0, >3.0), smoking status (never smoker, former smoker, current smoker), and alcohol drinking (non-drinker, low to moderate drinker, heavy drinker). Model 2: further adjusted (from Model 1) for chronic kidney disease (yes or no), diabetes mellitus (yes or no), and coronary heart disease (yes or no). Model 3: further adjusted (from Model 2) for eGFR (continuous), FBG (continuous), and HbA1c (continuous). Squares represent odds ratios and solid black lines indicate 95% confidence intervals. All estimates accounted for complex survey design. OR, odds ratio; CI, confidence interval; LDL-C, low-density lipoprotein cholesterol; RC, remnant cholesterol.

Compared to the low LDL-C/low RC group, the participants in low LDL-C/high RC group and high LDL-C/high RC group were associated with hypertension (low LDL-C/high RC group: OR, 2.04; 95% CI, 1.72-2.42; high LDL-C/high RC group: OR, 1.81; 95% CI, 1.55-2.10) (**Table 3**). This increase remained noticeable after adjusting for BMI, HDL-C or TG (model 1-3, **Supplemental Table S14**). On the other hand, those in the high LDL-C/low RC group had similar hypertension prevalence compared to the low LDL-C/low RC group. We found almost identical results when using different clinical cut-points to define concordance/discordance, except for the clinical cut-point of LDL-C 70 mg/dL and RC 13 mg/dL, given the smaller sample size and fewer number of positive events (**Figure 3**).

**Table 3:** Adjusted ORs (95%CIs) of hypertension according to different concordant/discordant groups ^*^.

	Concordant/discordant of LDL-C and RC, OR (95%CI)			
	Low LDL-C and low RC	Low LDL-C and high RC	High LDL-C and low RC	High LDL-C and high RC
Cases, No./Total No.	1,190/3,517	954/1,853	680/1,558	2,139/3,914
Model 1 ^†^	ref	2.18 (1.84-2.59)	1.04 (0.87-1.25)	1.85 (1.60-2.14)
Model 2 ^‡^	ref	2.07 (1.75-2.46)	1.08 (0.89-1.30)	1.83 (1.57-2.12)
Model 3 ^§^	ref	2.04 (1.72-2.42)	1.08 (0.90-1.31)	1.81 (1.55-2.10)

CI, confidence interval; LDL-C, low-density lipoprotein cholesterol; OR, odds ratio; RC, remnant cholesterol.

^*^ The different concordant/discordant groups based on LDL-C 118 mg/dL and RC 20 mg/dL cut-points.

^†^ Model1: adjusted for age (continuous), sex (male/female), race/ethnicity (non-Hispanic white, non-Hispanic black, Mexican American, other), educational level (less than high school, high school or equivalent, college or above), family income-poverty ratio (≤1.0, 1.1-3.0, >3.0), smoking status (never smoker, former smoker, current smoker), and alcohol drinking (non-drinker, low to moderate drinker, heavy drinker).

^‡^ Model 2: further adjusted (from Model 1) for chronic kidney disease (yes or no), diabetes mellitus (yes or no), and coronary heart disease (yes or no).

^§^ Model 3: further adjusted (from Model 2) for eGFR (continuous), FBG (continuous), and HbA1c (continuous).

All estimates accounted for complex survey design.

The association between tertiles of RC and hypertension was slightly weakened after adjusting for hs-CRP (N=1,947, model 4, **Supplemental Table S13**), but the significance of higher prevalence of hypertension was attenuated among individuals in low LDL-C/high RC group (model 4, **Supplemental Table S14**).

**Figure 4:**
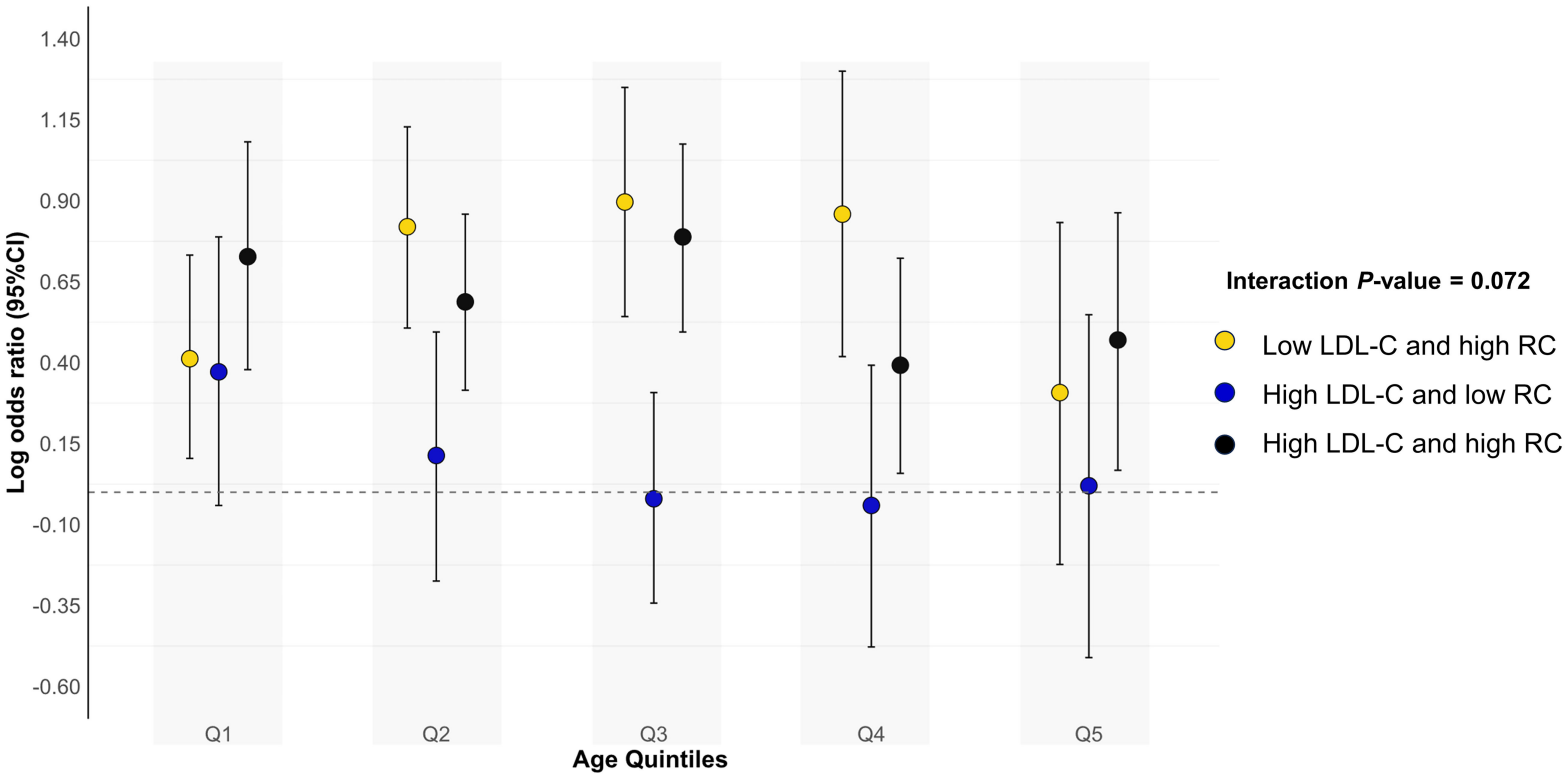
Associations (log odds ratios, 95%CIs) of different concordant/discordant groups with hypertension according to age quintiles. Low LDL-C and low RC was used as the reference group. Results were adjusted for age (continuous), sex (male/female), race/ethnicity (non-Hispanic white, non-Hispanic black, Mexican American, other), educational level (less than high school, high school or equivalent, college or above), family income-poverty ratio (≤1.0, 1.1-3.0, >3.0), smoking status (never smoker, former smoker, current smoker), alcohol drinking (non-drinker, low to moderate drinker, heavy drinker), chronic kidney disease (yes or no), diabetes mellitus (yes or no), coronary heart disease (yes or no), eGFR (continuous), FBG (continuous), and HbA1c (continuous). Circles represent log odds ratios and vertical lines indicate 95% confidence intervals. All estimates accounted for complex survey design. All estimates accounted for complex survey design. CI, confidence interval; LDL-C, low-density lipoprotein cholesterol; RC, remnant cholesterol.

Results were consistent when analyses were stratified by age quintiles, BMI, self-reported diabetes mellitus, self-reported chronic kidney disease, survey cycles and hypoglycemic drugs use (**Figure 4** and **Supplemental Table S15**). The test for interactions was significant for sex and smoking status (all *p*_interaction_ < 0.05), with low LDL-C/high RC more likely to be related to hypertension in males, never smokers and former smokers. Finally, we observed that our results did not materially change after excluding those previously informed of hypertension (N=322) or those taking antihypertensive drugs (N=682) (**Supplemental Tables S16 and S17**). Results were consistent when using Sampson-NIH formula or Friedewald equation to recalculate LDL-C and RC (**Supplemental Table S18**).

### Association between apoB and RC concordant/discordant groups with hypertension

A supplementary analysis (NHANES 2007-2016, N=6,854) showed that among participants with apoB < median (92mg/dL), those with discordant RC ≥ median (20mg/dL) had significantly higher odds of hypertension after adjusting potential confounders (OR, 1.73; 95% CI, 1.38–2.17) (**Table 4**).

**Table 4:** Adjusted ORs (95%CIs) of hypertension according to different concordant/discordant groups ^*^.

	Concordant/discordant of apoB and RC, OR (95%CI)			
	Low apoB and low RC	Low apoB and high RC	High apoB and low RC	High apoB and high RC
Cases, No./Total No.	773/2,405	406/919	411/945	1,337/2,585
Model 1 ^†^	Ref	1.82 (1.45-2.28)	1.13 (0.93-1.37)	1.95 (1.66-2.27)
Model 2 ^‡^	Ref	1.76 (1.40-2.22)	1.16 (0.95-1.42)	1.88 (1.61-2.20)
Model 3 ^§^	Ref	1.73 (1.38-2.17)	1.15 (0.94-1.41)	1.83 (1.55-2.15)

apoB, apolipoprotein B; CI, confidence interval; OR, odds ratio; RC, remnant cholesterol.

^*^ The different concordant/discordant groups based on apoB 92 mg/dL and RC 20 mg/dL cut-points in the NHANES 2007 to 2016.

^†^ Model1: adjusted for age (continuous), sex (male/female), race/ethnicity (non-Hispanic white, non-Hispanic black, Mexican American, other), educational level (less than high school, high school or equivalent, college or above), family income-poverty ratio (≤1.0, 1.1-3.0, >3.0), smoking status (never smoker, former smoker, current smoker), and alcohol drinking (non-drinker, low to moderate drinker, heavy drinker).

^‡^ Model 2: further adjusted (from Model 1) for chronic kidney disease (yes or no), diabetes mellitus (yes or no), and coronary heart disease (yes or no).

^§^ Model 3: further adjusted (from Model 2) for eGFR (continuous), FBG (continuous), and HbA1c (continuous).

All estimates accounted for complex survey design.

## Discussion

In a large and nationally representative sample of general US adults, we found that (i) elevated RC levels were associated with hypertension independent of multiple risk factors, including LDL-C levels and (ii) those with low LDL-C/high RC, but not high LDL-C/low RC, were significantly associated with hypertension independent of HDL-C and TG. Additional analyses showed that RC-associated high prevalence of hypertension may not be related to apoB. Our findings suggest that the association between RC and hypertension beyond LDL-C among the general population. Early identification of RC related risk, especially at normal LDL-C levels, may facilitate the differentiation of individuals at high risk of predisposing to hypertension.

Studies based on Chinese populations have shown that increased RC concentrations was linked to elevated blood pressure and the onset of hypertension (11, 22). Liu et al. (23) found that both RC and TG levels correlated more prominently with arterial stiffness (measured by brachial-ankle pulse wave velocity) than other lipid indices. Another study showed that RC was an independent predictor of endothelial dysfunction (reflected by flow-mediated vasodilation) in the general population (24). These suggest that the close association of RC with hypertension may be linked to poor vascular endothelial function and arterial elasticity. A study by Wu et al. (12) further found that the risk of advancement of arteriosclerosis and atherosclerosis was higher in individuals with high levels of RC, even when LDL-C was in the normal range. Reaffirming the above findings, we demonstrated here in large-scale US general population that RC levels were associated with hypertension in fully adjusted models including LDL-C. We further clarified the position of RC beyond LDL-C in the relationship between cholesterol and hypertension. Considering that hypertension is an important risk factor for ASCVD, our findings may provide evidence for the superior ability of RC to predict ASCVD risk over LDL-C shown in previous studies (6, 7).

Exogenous triglycerides are carried by apoB-48-containing chylomicrons, while hepatic-derived triglycerides are mainly released by apoB-100-containing very low-density lipoproteins (VLDL) particles. These TRLs undergo hydrolysis by lipoprotein lipase and decrease in size, in parallel with a decrease in TG content and an increase in cholesteryl esters in the presence of cholesteryl ester transfer protein, and thus forming small, dense cholesterol-rich remnants (25). One-third of non-fasting plasma cholesterol is present in remnant lipoproteins (26). Several reasons may explain the mechanism behind this association found in our study. First, elevated RC may act as a silent promoter of atherosclerotic cardiovascular disease (27). Apart from newly secreted chylomicrons and very large VLDL, most cholesterol-rich remnant particles below 70 nm diameter traverse the endothelium by active transcytosis and remain in the subendothelial layer of the arterial wall, where macrophages phagocytose them and form foam cells, promoting lesion occurrence and progression (28). These remnant particles share similar pathogenic mechanism to that of LDL, but they carry four times more cholesterol molecules per particle than LDL (29). In addition, their larger size compared to LDL, along with their enrichment in apolipoprotein C-III and apolipoprotein E, which increases their affinity for proteoglycans (30, 31), makes them more difficult to return to the arterial lumen. Apolipoprotein E also mediates subendothelial macrophage surface receptor-mediated endocytosis of remnants, which facilitates atherosclerosis (32). Second, increased RC levels may be associated with impaired endothelial function. The mechanism involves induction of apoptosis in endothelial cell through NAD(P)H oxidase-mediated formation of superoxide and production of tumor necrosis factor-α and interleukin 1β, and effects on nitric oxide synthase activity (33, 34). This is corroborated by the decrease of flow-mediated vasodilation indicator and the increase of brachial-ankle pulse wave velocity in previous studies. Third, the causal link between RC and low inflammation exacerbates atherosclerosis and endothelial impairment, whereas the pro-atherosclerotic effect of elevated LDL-C lacks the contribution of inflammation, and this causal association persists even in the absence of diabetes and obesity (35). It may explain the attenuated association of discordantly low LDL-C/high RC with hypertension after adjusting for hs-CRP. Forth, VLDL is catabolized into VLDL remnants, intermediate-density lipoproteins and LDL. Increased levels of VLDL may lead to accumulation of LDL, exerting multiple pathogenic effects.

Remnant particles formation is augmented by overproduction of TRLs or by genetic or physiological triggers that limit lipolysis of lipoprotein lipase, or both (29). In our continuous and tertiles analyses, we showed that increased RC levels were still associated with hypertension even after adjusting for TG. These observations were reproduced in concordant/discordant analyses. These findings suggest that elevated RC levels, regardless of TG levels, may indirectly reflected risk information related to downregulating lipolysis of lipoprotein lipase mechanisms such as decreased apolipoprotein C-II or apolipoprotein A-V activity and increased apolipoprotein C-III or angiopoietin-like protein 3 activity rather than the risk caused by increased remnants from elevated plasma TG levels (36, 37). It may also explain the increased odds of hypertension in those with high RC when apoB was below the threshold. As hypothesized in the study by Quispe et al. (6), RC predicted risk of cardiovascular disease independent of apoB partially explained by RC reflected the activity of key lipid regulatory proteins such as apolipoprotein C-III and angiopoietin-like protein 3, which may be independent of apoB contained on TRLs.

Disparities between the sexes in the stratified analyses may be related to the protection of the vascular endothelium by endogenous estrogen, and the differences in lipid metabolism and the amount and distribution of adipose tissue, with accumulated adipose tissue in different areas may play distinct pathological roles (38). Results stratified by smoking status need to be validated through further research as smoking impairs vascular endothelial function and promotes oxidative stress causing hypertension (39, 40). We speculate that better control of other risk factors not included in our study by current smokers may have contributed to this.

Unfortunately, there is no large-scale clinical study on reducing the risk of ASCVD in RC management. The relevant evidence was mainly found in subgroups of clinical studies or *post hoc* analyses. Previous studies have shown that statins, fibrates, and omega-3 fatty acids can lower RC levels, improve endothelial function, and regulate blood pressure (41–46). However, more evidence is needed on how much reduction in RC levels accompanies the benefits of blood pressure management. Novel therapies that target the metabolic modulation of TRLs may be promising in the future.

### Limitations

First, as this study is observational, we cannot rule out the potential of residual confounders. For example, the intake of some medications or lifestyle habits may affect blood pressure, and the consumption of certain supplements such as fish oil may have an effect on RC levels (46). In addition, we cannot draw causal inferences regarding elevated RC levels and hypertension due to the cross-sectional nature of the study design. Second, RC concentrations were not directly measured but estimated, giving rise to possible deviations from the actual levels. However, the calculated RC correlates favorably with the directly measured RC (R^2^ 0.69) (47) and can be obtained easily for practical application. Third, the association between hypertension and fasting RC levels in our study may be underestimated, considering that postprandial RC levels reflect more information. It remains to be investigated whether postprandial RC levels are also associated with hypertension beyond LDL-C in the US population. Forth, our study was limited to participants with TG ≤400 mg/dL. Derivation of the findings to the overall population will require additional studies to confirm. Fifth, our study included data from those who participated in NHANES from 1999-2018, spanning 20 years. Although subgroup analyses of the 1999-2008 and 2009-2018 survey cycles showed no significant differences in the results, changes in lifestyle, diet, and medication of individuals during different periods may still affect the results of this study.

### Conclusions

This study indicated that elevated RC levels were associated with hypertension independent of LDL-C in the general population. Clinical attention should be paid to RC levels in hypertensive individuals, especially in the absence of elevated LDL-C. In addition, this association extended beyond elevated serum TG levels, and lipoproteins other than apoB may be involved. The present study only illustrates a strong correlation, and the association between RC levels and the prognosis of hypertensive individuals remains to be studied.

## Data availability statement

Publicly available datasets were analyzed in this study. This data can be found here: https://wwwn.cdc.gov/nchs/nhanes/Default.aspx.

## Ethics statement

The studies involving humans were approved by National Center for Health Statistics (NCHS) ethics review board. The studies were conducted in accordance with the local legislation and institutional requirements. The participants provided their written informed consent to participate in this study.

## Author contributions

LS: Conceptualization, Methodology, Writing – original draft. DZ: Conceptualization, Writing – review and editing, Methodology. JJ: Data curation, Methodology, Writing – review and editing. AW: Data curation, Methodology, Writing – review and editing. TD: Data curation, Methodology, Writing – review and editing. XC: Writing – review and editing. YS: Writing – review and editing. ZG: Funding acquisition, Supervision, Writing – review and editing. HX: Funding acquisition, Supervision, Writing – review and editing.

## Supplementary material

Supplementary Figure 1 The distribution of LDL-C and RC by the concordant/discordant groups across LDL-C 118 mg/dL and RC 20 mg/dL cut-points. **(A)** The box plot of LDL-C and RC concentrations for the different concordant/discordant groups. **(B)** Proportions of LDL-C and RC concordance and discordance. Percentages were weighted. LDL-C, low-density lipoprotein cholesterol; RC, remnant cholesterol.
