# Supplementary material for: Remnant cholesterol associates with hypertension beyond low-density lipoprotein cholesterol among the general US adult population
Source: Front Endocrinol (Lausanne). 2023 Sep 29;14:1260764. doi: 10.3389/fendo.2023.1260764 (PMC10570462; doi:10.3389/fendo.2023.1260764)
Supplement: Supplementary file 1 [file DataSheet_1.docx]

**Supplemental Methods**

***Definitions and classifications of covariates***

Race/ethnicity was categorized as non-Hispanic White, non-Hispanic Black, Mexican American, or other. Family income-poverty ratio was classified as ≤ 1.0, 1.1-3.0, > 3.0. Education level was categorized as less than high school, high school or equivalent, or college or above. Smoking status was grouped into never, former, or current. History of diabetes, coronary heart disease, chronic kidney disease, stroke was self-reported. BMI was calculated as weight in kilograms divided by height in meters squared. Alcohol intake was classified as nondrinker, low to moderate drinker (defined as ≤2 drinks/day in men and ≤1 drink/day in women), or heavy drinker (defined as > 2 drinks/day in men and > 1 drink/day in women). Estimated glomerular filtration rate (eGFR) was calculated by using the Chronic Kidney Disease Epidemiology Collaboration formula.
