# Supplementary material for: Remnant cholesterol associates with hypertension beyond low-density lipoprotein cholesterol among the general US adult population
Source: Front Endocrinol (Lausanne). 2023 Sep 29;14:1260764. doi: 10.3389/fendo.2023.1260764 (PMC10570462; doi:10.3389/fendo.2023.1260764)
Supplement: Supplementary file 2 [file Table_1.doc]

**SUPPLEMENTARY TABLES**

**Remnant cholesterol associates with hypertension beyond low-density lipoprotein cholesterol among the general US adult population**

**Supplemental Tables**

***Table S1*** Collinearity diagnostics of log RC and log LDL-C with adjusted covariates

|  | Variance inflation factor |
| --- | --- |
|  | Step 1 |
| log RC | 1.6 |
| log LDL-C | 1.7 |
| Age | 2.5 |
| eGFR | 2.4 |
| FBG | 3.6 |
| HbA1c | 3.5 |
| Sex | 1.4 |
| Race/ethnicity | 2.4 |
| Family income-poverty ratio | 1.7 |
| Educational level | 1.6 |
| Smoking status | 1.6 |
| Alcohol drinking | 2.0 |
| Chronic kidney disease | 1.2 |
| Diabetes mellitus | 2.1 |
| Coronary heart disease | 1.3 |

Abbreviations as in Table ​1.

Note: Variance inflation factor = 1/(1-R^2^).

***Table S2*** Collinearity diagnostics of RC tertiles with adjusted covariates

|  | Variance inflation factor |
| --- | --- |
|  | Step 1 |
| RC tertiles | 1.7 |
| log LDL-C | 1.7 |
| Age | 2.6 |
| eGFR | 2.4 |
| FBG | 3.6 |
| HbA1c | 3.4 |
| Sex | 1.4 |
| Race/ethnicity | 2.4 |
| Family income-poverty ratio | 1.9 |
| Educational level | 1.6 |
| Smoking status | 1.6 |
| Alcohol drinking | 2.1 |
| Chronic kidney disease | 1.2 |
| Diabetes mellitus | 2.1 |
| Coronary heart disease | 1.2 |

Abbreviations as in Table ​1.

Note: Variance inflation factor = 1/(1-R^2^).

***Table S3*** Collinearity diagnostics of LDL-C tertiles with adjusted covariates

|  | Variance inflation factor |
| --- | --- |
|  | Step 1 |
| LDL-C tertiles | 2.0 |
| log RC | 1.7 |
| Age | 2.7 |
| eGFR | 2.4 |
| FBG | 3.6 |
| HbA1c | 3.4 |
| Sex | 1.4 |
| Race/ethnicity | 2.5 |
| Family income-poverty ratio | 1.7 |
| Educational level | 1.6 |
| Smoking status | 1.6 |
| Alcohol drinking | 2.1 |
| Chronic kidney disease | 1.2 |
| Diabetes mellitus | 2.1 |
| Coronary heart disease | 1.3 |

Abbreviations as in Table ​1.

Note: Variance inflation factor = 1/(1-R^2^).

***Table S4*** Collinearity diagnostics of LDL-C and RC concordant/discordant groups with adjusted covariates

|  | Variance inflation factor |
| --- | --- |
|  | Step 1 |
| Concordant/discordant groups of LDL-C and RC | 2.4 |
| Age | 2.6 |
| eGFR | 2.5 |
| FBG | 3.4 |
| HbA1c | 3.2 |
| Sex | 1.4 |
| Race/ethnicity | 2.3 |
| Family income-poverty ratio | 1.6 |
| Educational level | 1.6 |
| Smoking status | 1.6 |
| Alcohol drinking | 2.1 |
| Chronic kidney disease | 1.3 |
| Diabetes mellitus | 2.2 |
| Coronary heart disease | 1.3 |

Abbreviations as in Table ​1.

Note: Variance inflation factor = 1/(1-R^2^).

***Table S5*** Collinearity diagnostics of RC tertiles with further adjusted covariates

|  | Variance inflation factor | |
| --- | --- | --- |
|  | Step 1 | Step 2 |
| RC tertiles | **5.1** | 4.9 |
| **BMI** | **7.2** | 1.5 |
| **WC** | **7.2** | **-** |
| **HDL-C** | 1.7 | 1.6 |
| **TG** | 4.3 | 4.1 |
| Age | 2.6 | 2.4 |
| eGFR | 2.5 | 2.4 |
| FBG | 4.8 | 4.7 |
| HbA1c | 4.6 | 4.5 |
| Sex | 1.6 | 1.5 |
| Race/ethnicity | 3.0 | 2.7 |
| Family income-poverty ratio | 2.0 | 2.0 |
| Educational level | 1.9 | 1.7 |
| Smoking status | 1.9 | 1.8 |
| Alcohol drinking | 2.2 | 2.2 |
| Chronic kidney disease | 1.3 | 1.2 |
| Diabetes mellitus | 2.3 | 2.2 |
| Coronary heart disease | 1.3 | 1.3 |

Abbreviations as in Table ​1.

Note: Variance inflation factor = 1/(1-R^2^).

***Table S6*** Collinearity diagnostics of LDL-C tertiles with further adjusted covariates

|  | Variance inflation factor | |
| --- | --- | --- |
|  | Step 1 | Step 2 |
| LDL-C tertiles | 2.1 | 2.0 |
| **BMI** | **7.3** | 1.5 |
| **WC** | **7.5** | **-** |
| **HDL-C** | 1.7 | 1.6 |
| **TG** | 1.7 | 1.6 |
| Age | 2.8 | 2.6 |
| eGFR | 2.5 | 2.4 |
| FBG | 4.8 | 4.7 |
| HbA1c | 4.8 | 4.7 |
| Sex | 1.7 | 1.5 |
| Race/ethnicity | 3.1 | 2.7 |
| Family income-poverty ratio | 1.8 | 1.8 |
| Educational level | 1.9 | 1.7 |
| Smoking status | 1.9 | 1.8 |
| Alcohol drinking | 2.1 | 2.1 |
| Chronic kidney disease | 1.2 | 1.2 |
| Diabetes mellitus | 2.3 | 2.3 |
| Coronary heart disease | 1.4 | 1.3 |

Abbreviations as in Table ​1.

Note: Variance inflation factor = 1/(1-R^2^).

***Table S7*** Collinearity diagnostics of LDL-C and RC concordant/discordant groups with further adjusted covariates

|  | Variance inflation factor | |
| --- | --- | --- |
|  | Step 1 | Step 2 |
| Concordant/discordant groups of LDL-C and RC | **5.6** | **5.4** |
| **BMI** | **7.7** | 1.6 |
| **WC** | **7.9** | - |
| **HDL-C** | 1.7 | 1.7 |
| **TG** | 2.8 | 2.6 |
| Age | 2.7 | 2.5 |
| eGFR | 2.5 | 2.5 |
| FBG | 4.8 | 4.7 |
| HbA1c | 4.7 | 4.6 |
| Sex | 1.7 | 1.5 |
| Race/ethnicity | 3.1 | 2.8 |
| Family income-poverty ratio | 1.9 | 1.9 |
| Educational level | 1.9 | 1.7 |
| Smoking status | 1.9 | 1.8 |
| Alcohol drinking | 2.2 | 2.2 |
| Chronic kidney disease | 1.3 | 1.3 |
| Diabetes mellitus | 2.4 | 2.4 |
| Coronary heart disease | 1.4 | 1.3 |

Abbreviations as in Table ​1.

Note: Variance inflation factor = 1/(1-R^2^).

***Table S8*** Collinearity diagnostics of RC tertiles and with further adjusted covariates

|  | Variance inflation factor | | |
| --- | --- | --- | --- |
|  | Step 1 | Step 2 | Step 3 |
| RC tertiles | **14.7** | 3.1 | 2.1 |
| **hs-CRP** | 3.5 | 1.8 | 1.6 |
| Age | **15.0** | 4.4 | 3.0 |
| eGFR | **26.0** | 4.4 | 3.9 |
| FBG | **17.4** | **6.4** | - |
| HbA1c | **20.5** | **5.6** | 2.1 |
| Sex | 3.5 | 2.1 | 1.4 |
| Race/ethnicity | **88.8** | - | - |
| Family income-poverty ratio | **39.9** | - | - |
| Educational level | **33.8** | - | - |
| Smoking status | **22.2** | **6.9** | - |
| Alcohol drinking | **12.1** | 3.2 | 1.9 |
| Chronic kidney disease | 2.3 | 1.6 | 1.7 |
| Diabetes mellitus | 4.7 | 3.2 | 1.8 |
| Coronary heart disease | 3.7 | 1.8 | 1.6 |

Abbreviations as in Table ​1.

Note: Variance inflation factor = 1/(1-R^2^).

***Table S9*** Collinearity diagnostics of LDL-C tertiles and with further adjusted covariates

|  | Variance inflation factor | |
| --- | --- | --- |
|  | Step 1 | Step 2 |
| LDL-C tertiles | **12.9** | 5.0 |
| **hs-CRP** | 2.7 | 1.8 |
| Age | **12.0** | 3.5 |
| eGFR | **24.4** | - |
| FBG | **16.1** | 4.5 |
| HbA1c | **14.5** | 3.8 |
| Sex | 5.0 | 2.3 |
| Race/ethnicity | **104.4** | - |
| Family income-poverty ratio | **22.3** | - |
| Educational level | **18.5** | - |
| Smoking status | **16.3** | 3.7 |
| Alcohol drinking | **8.6** | 3.1 |
| Chronic kidney disease | 2.5 | 1.3 |
| Diabetes mellitus | **4.6** | 3.3 |
| Coronary heart disease | **4.5** | 2.3 |

Abbreviations as in Table ​1.

Note: Variance inflation factor = 1/(1-R^2^).

***Table S10*** Collinearity diagnostics of LDL-C and RC concordant/discordant groups with further adjusted covariates

|  | Variance inflation factor | | |
| --- | --- | --- | --- |
|  | Step 1 | Step 2 | Step3 |
| Concordant/discordant groups of LDL-C and RC | **53.5** | **11.9** | **9.4** |
| **hs-CRP** | 3.4 | 1.8 | 1.6 |
| Age | **10.1** | 4.7 | 3.0 |
| eGFR | **16.6** | **5.7** | - |
| FBG | **16.0** | **6.4** | - |
| HbA1c | **15.2** | 4.7 | 2.3 |
| Sex | 4.9 | 2.0 | 1.5 |
| Race/ethnicity | **86.8** | - | - |
| Family income-poverty ratio | **23.8** | - | - |
| Educational level | **29.0** | - | - |
| Smoking status | **16.8** | - | - |
| Alcohol drinking | **12.4** | 2.4 | 2.1 |
| Chronic kidney disease | 2.8 | 1.9 | 1.4 |
| Diabetes mellitus | **6.0** | 4.1 | 3.3 |
| Coronary heart disease | 4.2 | 2.1 | 2.2 |

Abbreviations as in Table ​1.

Note: Variance inflation factor = 1/(1-R^2^).

***Table S11* Characteristics of participants by hypertension status ^*^**

| **Characteristic** | **Total**  **(n=10842)** | **Hypertension**  **(n = 4963)** | **Non-hypertension**  **(n = 5879)** | ***P* value** |
| --- | --- | --- | --- | --- |
| Age, (mean ± SE), years | 42.20 ± 0.21 | 48.34 ± 0.28 | 37.84 ± 0.24 | <0.001 |
| Sex |  |  |  |  |
| Male | 5048 (48.17) | 2076 (42.75) | 2972 (52.02) | <0.001 |
| Female | 5794 (51.83) | 2887 (57.25) | 2907 (47.98) |
| Race/ethnicity |  |  |  |  |
| Non-Hispanic White | 4909 (69.31) | 2227 (69.62) | 2682 (69.09) | <0.001 |
| Non-Hispanic Black | 2103 (10.71) | 1191 (13.58) | 912 (8.67) |
| Mexican American | 2065 (8.72) | 859 (7.10) | 1206 (9.87) |
| Other | 1765 (11.26) | 686 (9.70) | 1079 (12.37) |
| Educational level |  |  |  |  |
| Less than high school | 985 (4.32) | 540 (5.05) | 445 (3.81) | <0.001 |
| High school or equivalent | 4007 (34.04) | 1980 (37.27) | 2027 (31.75) |
| College or above | 5850 (61.64) | 2443 (57.68) | 3407 (64.44) |
| Family income-poverty ratio |  |  |  |  |
| ≤1.0 | 2083 (13.09) | 935 (12.79) | 1148 (13.30) | 0.769 |
| 1.1-3.0 | 4420 (35.56) | 2077 (35.82) | 2343 (35.38) |
| >3.0 | 4339 (51.35) | 1951 (51.38) | 2388 (51.33) |
| Smoking status |  |  |  |  |
| Never smoker | 5581 (51.52) | 2378 (49.00) | 3203 (53.30) | <0.001 |
| Former smoker | 2593 (23.84) | 1421 (27.56) | 1172 (21.20) |
| Current smoker | 2668 (24.64) | 1164 (23.44) | 1504 (25.50) |
| Alcohol drinking |  |  |  |  |
| Non-drinker | 1859 (13.90) | 1069 (17.52) | 790 (11.32) | <0.001 |
| Low to moderate drinker | 4195 (41.00) | 1803 (38.87) | 2392 (42.51) |
| Heavy drinker | 4788 (45.10) | 2091 (43.61) | 2697 (46.16) |
| BMI | 28.32 ± 0.10 | 30.28 ± 0.15 | 26.93 ± 0.12 | <0.001 |
| Waist Circumference | 96.82 ± 0.26 | 102.75 ± 0.35 | 92.61 ± 0.30 | <0.001 |
| SBP, mmHg | 119.46 ± 0.21 | 130.70 ± 0.31 | 111.47 ± 0.18 | <0.001 |
| DBP, mmHg | 70.98 ± 0.18 | 77.06 ± 0.25 | 66.67 ± 0.16 | <0.001 |
| Diabetes mellitus | 1141 (7.36) | 849 (12.81) | 292 (3.48) | <0.001 |
| Coronary heart disease | 127 (0.72) | 102 (1.33) | 25 (0.29) | <0.001 |
| Chronic kidney disease | 1258 (8.84) | 937 (14.44) | 321 (4.85) | <0.001 |
| Stroke | 184 (1.18) | 136 (2.00) | 48 (0.59) | <0.001 |
| Antihypertensive drugs use | 682 (5.23) | 682 (12.60) | 0 (0.00) | <0.001 |
| Hypoglycemic drugs use | 454 (2.84) | 348 (5.04) | 106 (1.27) | <0.001 |
| LDL-C, (mean ± SE), mg/dL | 119.97 ± 0.46 | 126.31 ± 0.65 | 115.46 ± 0.60 | <0.001 |
| HDL-C, (mean ± SE), mg/dL | 53.93 ± 0.24 | 52.67 ± 0.30 | 54.83 ± 0.32 | <0.001 |
| Non-HDL-C, (mean ± SE), mg/dL | 142.40 ± 0.53 | 150.90 ± 0.74 | 136.33 ± 0.70 | <0.001 |
| TG, (mean ± SE), mg/dL | 117.53 ± 0.87 | 133.40 ± 1.3 | 106.23 ± 1.09 | <0.001 |
| TC, (mean ± SE), mg/dL | 196.33 ± 0.54 | 203.60 ± 0.74 | 191.16 ± 0.70 | <0.001 |
| RC, (mean ± SE), mg/dL | 22.44 ± 0.12 | 24.64 ± 0.17 | 20.87 ± 0.15 | <0.001 |
| FBG, (mean ± SE), mmol/L | 5.59 ± 0.01 | 5.87 ± 0.03 | 5.39 ± 0.02 | <0.001 |
| HbA1c, (mean ± SE), % | 5.41 ± 0.01 | 5.56 ± 0.01 | 5.30 ± 0.01 | <0.001 |
| eGFR, (mean ± SE), mL/min/1.73 m^2^ | 99.00 ± 0.30 | 93.68 ± 0.38 | 102.79 ± 0.37 | <0.001 |
| ApoB, mg/dL ^†^ | 93.45 ± 0.52 | 99.52 ± 0.69 | 89.60 ± 0.58 | <0.001 |
| Hs-CRP, mg/L ^‡^ | 3.63 ± 0.21 | 4.67 ± 0.38 | 2.88 ± 0.20 | <0.001 |
| **Discordant/concordant of LDL-C and RC** | | | | |
| Low LDL-C and low RC | 3517 (33.65) | 1190 (23.36) | 2327 (40.96) | <0.001 |
| Low LDL-C and high RC | 1853 (16.67) | 954 (19.55) | 899 (14.62) |
| High LDL-C and low RC | 1558 (14.19) | 680 (13.04) | 878 (15.01) |
| High LDL-C and high RC | 3914 (35.49) | 2139 (44.05) | 1775 (29.41) |
| Abbreviations: apoB, apolipoprotein B; BMI, body mass index; DBP, diastolic blood pressure; eGFR, estimated glomerular filtration rate; FBG, fasting blood glucose; HbA1c, glycosylated hemoglobin A1c; hs-CRP, high-sensitivity C-reactive protein HDL-C, high-density lipoprotein cholesterol; LDL-C, low-density lipoprotein cholesterol; RC, remnant cholesterol; SBP, systolic blood pressure; TG, triglycerides; TC, total cholesterol.  SI conversions: to convert LDL-C, HDL-C, Non-HDL-C, TC, and RC to mmol/L, multiply by 0.02586; to convert TG to mmol/L, multiply by 0.01129.  ^*^ All means and SEs for continuous variables and percentages for categorical variables were weighted, with the exception of the number of participants.  ^†^ ApoB data only available in 2007-2016.  ^‡^ hs-CRP data only available in 2015-2018. | | | | |

***Table S12* Characteristics of** **participants According to Tertiles of RC levels ^*^**

| **Characteristic** | **Serum RC Concentrations (mg/dL)** | | | ***P* value** |
| --- | --- | --- | --- | --- |
|  | **Tertile 1**  **(n=3615)** | **Tertile 2**  **(n=3602)** | **Tertile 3**  **(n=3625)** |  |
| Age, (mean ± SE), years | 42.20 ± 0.21 | 38.96 ± 0.32 | 45.23 ± 0.27 | <0.001 |
| Sex |  |  |  |  |
| Male | 2033 (57.95) | 1610 (45.95) | 1405 (40.12) | <0.001 |
| Female | 1582 (42.05) | 1992 (54.05) | 2220 (59.88) |  |
| Race/ethnicity |  |  |  |  |
| Non-Hispanic White | 1517 (66.31) | 1657 (69.92) | 1735 (71.85) | <0.001 |
| Non-Hispanic Black | 1023 (15.76) | 685 (10.19) | 395 (5.92) |  |
| Mexican American | 476 (6.85) | 700 (9.19) | 889 (10.21) |  |
| Other | 599 (11.08) | 560 (10.70) | 606 (12.01) |  |
| Educational level |  |  |  |  |
| Less than high school | 190 (2.73) | 322 (4.43) | 473 (5.90) | <0.001 |
| High school or equivalent | 1204 (29.26) | 1355 (34.93) | 1448 (38.18) |  |
| College or above | 2221 (68.01) | 1925 (60.65) | 1704 (55.93) |  |
| Family income-poverty ratio |  |  |  |  |
| ≤1.0 | 680 (12.84) | 673 (12.93) | 730 (13.51) | 0.067 |
| 1.1-3.0 | 1387 (33.65) | 1517 (36.82) | 1516 (36.31) |  |
| >3.0 | 1548 (53.51) | 1412 (50.26) | 1379 (50.19) |  |
| Smoking status |  |  |  |  |
| Never smoker | 2123 (58.31) | 1824 (50.17) | 1634 (45.74) | <0.001 |
| Former smoker | 747 (21.06) | 842 (23.96) | 1004 (26.65) |  |
| Current smoker | 745 (20.63) | 936 (25.87) | 987 (27.62) |  |
| Alcohol drinking |  |  |  |  |
| Non-drinker | 507 (11.54) | 610 (13.72) | 742 (16.56) | <0.001 |
| Low to moderate drinker | 1679 (47.84) | 1355 (39.84) | 1161 (34.97) |  |
| Heavy drinker | 1429 (40.62) | 1637 (46.44) | 1722 (48.47) |  |
| BMI | 26.31 ± 0.16 | 28.50 ± 0.14 | 30.26 ± 0.17 | <0.001 |
| Waist Circumference | 90.40 ± 0.39 | 97.50 ± 0.35 | 102.90 ± 0.41 | <0.001 |
| SBP, mmHg | 115.83 ± 0.33 | 119.50 ± 0.27 | 123.24 ± 0.35 | <0.001 |
| DBP, mmHg | 68.38 ± 0.25 | 71.07 ± 0.26 | 73.63 ± 0.24 | <0.001 |
| Hypertension | 1238 (29.46) | 1667 (41.52) | 2058 (54.27) | <0.001 |
| Diabetes mellitus | 212 (3.94) | 364 (6.80) | 565 (11.53) | <0.001 |
| Coronary heart disease | 26 (0.42) | 44 (0.76) | 57 (0.99) | 0.011 |
| Chronic kidney disease | 300 (6.61) | 422 (8.73) | 536 (11.29) | <0.001 |
| Stroke | 43 (0.68) | 67 (1.39) | 74 (1.50) | 0.003 |
| Antihypertensive drugs use | 142 (3.18) | 229 (5.10) | 311(7.54) | <0.001 |
| Hypoglycemic drugs use | 79 (1.34) | 166 (2.95) | 209 (4.30) | <0.001 |
| LDL-C, (mean ± SE), mg/dL | 98.98 ± 0.58 | 121.92 ± 0.69 | 140.09 ± 0.66 | <0.001 |
| HDL-C, (mean ± SE), mg/dL | 61.97 ± 0.38 | 53.61 ± 0.30 | 45.79 ± 0.30 | <0.001 |
| Non-HDL-C, (mean ± SE), mg/dL | 113.33 ± 0.60 | 142.57 ± 0.70 | 172.85 ± 0.72 | <0.001 |
| TG, (mean ± SE), mg/dL | 59.65 ± 0.34 | 103.40 ± 0.32 | 192.77 ± 1.28 | <0.001 |
| TC, (mean ± SE), mg/dL | 175.30 ± 0.70 | 196.18 ± 0.79 | 218.64 ± 0.78 | <0.001 |
| FBG, (mean ± SE), mmol/L | 5.34 ± 0.02 | 5.58 ± 0.02 | 5.88 ± 0.03 | <0.001 |
| HbA1c, (mean ± SE), % | 5.28 ± 0.01 | 5.40 ± 0.01 | 5.57 ± 0.02 | <0.001 |
| eGFR, (mean ± SE), mL/min/1.73 m^2^ | 102.58 ± 0.50 | 98.23 ± 0.37 | 96.03 ± 0.43 | <0.001 |
| ApoB, mg/dL ^†^ | 77.00 ± 0.50 | 93.50 ± 0.62 | 112.26 ± 0.78 | <0.001 |
| Hs-CRP, mg/L ^‡^ | 3.19 ± 0.32 | 4.31 ± 0.30 | 3.63 ± 0.34 | 0.032 |
| Abbreviations: apoB, apolipoprotein B; BMI, body mass index; DBP, diastolic blood pressure; eGFR, estimated glomerular filtration rate; FBG, fasting blood glucose; HbA1c, glycosylated hemoglobin A1c; hs-CRP, high-sensitivity C-reactive protein HDL-C, high-density lipoprotein cholesterol; LDL-C, low-density lipoprotein cholesterol; RC, remnant cholesterol; SBP, systolic blood pressure; TG, triglycerides; TC, total cholesterol.  SI conversions: to convert LDL-C, HDL-C, Non-HDL-C, TC, and RC to mmol/L, multiply by 0.02586; to convert TG to mmol/L, multiply by 0.01129.  ^*^ All means and SEs for continuous variables and percentages for categorical variables were weighted, with the exception of the number of participants.  ^†^ ApoB data only available in 2007-2016.  ^‡^ hs-CRP data only available in 2015-2018. | | | | |

***Table S13*** Adjusted ORs (95%CIs) of hypertension according to LDL-C and RC concentrations with further adjustment

| Variables | LDL-C and RC Levels, OR (95%CI) | | | |
| --- | --- | --- | --- | --- |
|  | Tertile1 | Tertile2 | Tertile3 | *P* _trend_ |
| LDL-C | | | | |
| Model 1^*^ | Ref | 1. 07 (0.93-1.23) | 1.13 (0.96-1.33) | 0.143 |
| Model 2^†^ | Ref | 1.17 (1.02-1.34) | 1.24 (1.05-1.45) | 0.011 |
| Model 3^‡^ | Ref | 1.09 (0.95-1.24) | 1.05 (0.89-1.24) | 0.585 |
| Model 4 ^§^ | Ref | 1.17 (0.84-1.63) | 1.39 (0.89-2.19) | 0.141 |
| RC | | | | |
| Model 1^*^ | Ref | 1.24 (1.08-1.43) | 1.68 (1.45-1.95) | <0.001 |
| Model 2^†^ | Ref | 1.41 (1.22-1.61) | 2.06 (1.76-2.41) | <0.001 |
| Model 3^‡^ | Ref | 1.26 (1.08-1.45) | 1.40 (1.10-1.78) | 0.009 |
| Model 4 || | Ref | 1.20 (0.88-1.66) | 2.02 (1.44-2.85) | <0.001 |
| Abbreviations: OR, odds ratio; CI, confidence interval; LDL-C, low-density lipoprotein cholesterol; RC, remnant cholesterol.  ^*^ Model 1: adjusted for age + sex + race/ethnicity + educational level + family income-poverty ratio + smoking status + alcohol drinking + chronic kidney disease + coronary heart disease + diabetes mellitus + eGFR + FBG + HbA1c + **BMI**.  ^†^ Model 2: adjusted for age + sex + race/ethnicity + educational level + family income-poverty ratio + smoking status + alcohol drinking + chronic kidney disease + coronary heart disease + diabetes mellitus + eGFR + FBG + HbA1c + **HDL-C**.  ^‡^ Model 3: adjusted for age + sex + race/ethnicity + educational level + family income-poverty ratio + smoking status + alcohol drinking + chronic kidney disease + coronary heart disease + diabetes mellitus + eGFR + FBG + HbA1c + **TG**.  ^§^ Model 4: adjusted for age + sex + smoking status + alcohol drinking + chronic kidney disease + coronary heart disease + diabetes mellitus + FBG + HbA1c + **hs-CRP**. hs-CRP data only available in 2015-2018.  || Model 4: adjusted for age + sex + alcohol drinking + chronic kidney disease + coronary heart disease + diabetes mellitus + eGFR + HbA1c + **hs-CRP**. hs-CRP data only available in 2015-2018.  All estimates accounted for complex survey design. | | | | |

***Table S14*** Adjusted ORs (95%CIs) of hypertension according to different concordant/discordant groups across LDL-C 118 mg/dL and RC 20 mg/dL cut-points with further adjustment

|  | Concordant/discordant of LDL-C and RC, OR (95%CI) | | | |
| --- | --- | --- | --- | --- |
|  | **Low LDL-C and low RC** | **Low LDL-C and high RC** | **High LDL-C and low RC** | **High LDL-C and high RC** |
| Model 1^*^ | Ref | 1.64 (1.38-1.95) | 0.98 (0.81-1.19) | 1.42 (1.22-1.67) |
| Model 2^†^ | Ref | 1.93 (1.62-2.29) | 1.08 (0.89-1.30) | 1.70 (1.44-2.01) |
| Model 3^‡^ | Ref | 1.48 (1.20-1.82) | 1.07 (0.88-1.29) | 1.30 (1.07-1.58) |
| Model 4 ^§^ | Ref | 1.28 (0.80-2.05) | 0.87 (0.52-1.48) | 1.72 (1.07-2.78) |
| Abbreviations: OR, odds ratio; CI, confidence interval; LDL-C, low-density lipoprotein cholesterol; RC, remnant cholesterol.  ^*^ Model 1: adjusted for age + sex + race/ethnicity + educational level + family income-poverty ratio + smoking status + alcohol drinking + chronic kidney disease + coronary heart disease + diabetes mellitus + eGFR + FBG + HbA1c + **BMI**.  ^†^ Model 2: adjusted for age + sex + race/ethnicity + educational level + family income-poverty ratio + smoking status + alcohol drinking + chronic kidney disease + coronary heart disease + diabetes mellitus + eGFR + FBG + HbA1c + **HDL-C**.  ^‡^ Model 3: adjusted for age + sex + race/ethnicity + educational level + family income-poverty ratio + smoking status + alcohol drinking + chronic kidney disease + coronary heart disease + diabetes mellitus + eGFR + FBG + HbA1c + **TG**.  ^§^ Model 4: adjusted for age + sex + alcohol drinking + chronic kidney disease + coronary heart disease + diabetes mellitus + HbA1c +**hs-CRP**. hs-CRP data only available in 2015-2018.  All estimates accounted for complex survey design. | | | | |

***Table S15*** Stratified analyses of the associations (odds ratios, 95%CIs) between hypertension and different concordant/discordant groups across LDL-C 118 mg/dL and RC 20 mg/dL cut-points in the NHANES 1999 to 2018

|  | **Concordant/discordant of LDL-C and RC, OR (95%CI)** | | | | |
| --- | --- | --- | --- | --- | --- |
|  | **Low LDL-C and low RC** | **Low LDL-C and high RC** | **High LDL-C and low RC** | **High LDL-C and high RC** | ***P*** **_interaction_** |
| **Sex** | | | | | 0.049 |
| Male | Ref | 2.34 (1.79-3.06) | 1.14 (0.89-1.48) | 2.05 (1.64-2.57) |  |
| Female | Ref | 1.73 (1.37-2.19) | 0.98 (0.74-1.30) | 1.54 (1.25-1.89) |  |
| **BMI (kg/m^2^)** | | | | | 0.053 |
| ＜25 | Ref | 2.06 (1.53-2.78) | 1.12 (0.79-1.59) | 1.38 (1.02-1.86) |  |
| 25-29.9 | Ref | 1.43 (1.06-1.92) | 0.90 (0.68-1.20) | 1.32 (1.02-1.70) |  |
| ≥30 | Ref | 1.69 (1.29-2.21) | 0.92 (0.66-1.29) | 1.52 (1.18-1.95) |  |
| **Smoking status** | | | | | 0.018 |
| Never smoker | Ref | 2.26 (1.81-2.82) | 1.14 (0.90-1.45) | 2.10 (1.67-2.63) |  |
| Former smoker | Ref | 2.22 (1.55-3.18) | 1.10 (0.73-1.66) | 1.98 (1.50-2.61) |  |
| Current smoker | Ref | 1.47 (1.08-2.01) | 0.90 (0.62-1.30) | 1.21 (0.92-1.60) |  |
| **Chronic kidney disease** | | | | | 0.255 |
| Yes | Ref | 2.60 (1.47-4.61) | 1.77 (1.00-3.14) | 1.78 (1.13-2.82) |  |
| No | Ref | 2.01 (1.68-2.40) | 1.05 (0.86-1.29) | 1.81 (1.55-2.12) |  |
| **Diabetes mellitus** | | | | | 0.090 |
| Yes | Ref | 1.76 (0.92-3.37) | 0.78 (0.36-1.67) | 1.06 (0.63-1.79) |  |
| No | Ref | 1.97 (1.66-2.35) | 1.07 (0.88-1.30) | 1.75 (1.50-2.05) |  |
| **Survey cycles** | | | | | 0.066 |
| 1999-2008 | Ref | 2.43 (1.93-3.06) | 1.17 (0.92-1.49) | 1.78 (1.46-2.16) |  |
| 2009-2018 | Ref | 1.61 (1.25-2.08) | 1.03 (0.77-1.37) | 1.85 (1.47-2.33) |  |
| **Hypoglycemic drugs use** |  |  |  |  | 0.545 |
| Yes^*^ | Ref | 2.09 (0.67-6.50) | 0.80 (0.21-3.02) | 1.14 (0.42-3.10) |  |
| No | Ref | 1.99 (1.68-2.36) | 1.07 (0.88-1.30) | 1.77 (1.52-2.07) |  |
| Abbreviations: OR, odds ratio; CI, confidence interval; LDL-C, low-density lipoprotein cholesterol; RC, remnant cholesterol; BMI, body mass index.  Results were adjusted for age (continuous), sex (male/female), race/ethnicity (non-Hispanic white, non-Hispanic black, Mexican American, other), educational level (less than high school, high school or equivalent, college or above), family income-poverty ratio (≤1.0, 1.1-3.0, >3.0), smoking status (never smoker, former smoker, current smoker), alcohol drinking (non-drinker, low to moderate drinker, heavy drinker), chronic kidney disease (yes or no), diabetes mellitus (yes or no), coronary heart disease (yes or no), eGFR (continuous), FBG (continuous), and HbA1c (continuous).  ^*^ Considering the stability of the model, diabetes mellitus (yes or no) was not adjusted when the subgroup was taking hypoglycemic medication.  All estimates accounted for complex survey design. | | | | | |

***Table S16*** Adjusted ORs (95%CIs) of hypertension according to different concordant/discordant groups across LDL-C 118 mg/dL and RC 20 mg/dL cut-points in the NHANES 1999 to 2018 excluding those previously informed of hypertension (n=322)

|  | Concordant/discordant of LDL-C and RC, OR (95%CI) | | | |
| --- | --- | --- | --- | --- |
|  | **Low LDL-C and low RC** | **Low LDL-C and high RC** | **High LDL-C and low RC** | **High LDL-C and high RC** |
| Model 1^*^ | Ref | 2.28 (1.90-2.72) | 1.05 (0.87-1.26) | 1.94 (1.66-2.27) |
| Model 2^†^ | Ref | 2.15 (1.80-2.58) | 1.08 (0.90-1.31) | 1.92 (1.64-2.25) |
| Model 3^‡^ | Ref | 2.11 (1.77-2.53) | 1.09 (0.90-1.32) | 1.89 (1.61-2.23) |
| Abbreviations: OR, odds ratio; CI, confidence interval; LDL-C, low-density lipoprotein cholesterol; RC, remnant cholesterol.  ^*^ Model 1: adjusted for age + sex + race/ethnicity + educational level + family income-poverty ratio + smoking status + alcohol drinking.  ^†^ Model 2: adjusted for age + sex + race/ethnicity + educational level + family income-poverty ratio + smoking status + alcohol drinking + chronic kidney disease + coronary heart disease + diabetes mellitus.  ^‡^ Model 3: adjusted for age + sex + race/ethnicity + educational level + family income-poverty ratio + smoking status + alcohol drinking + chronic kidney disease + coronary heart disease + diabetes mellitus + eGFR + FBG + HbA1c.  All estimates accounted for complex survey design. | | | | |

***Table S17*** Adjusted ORs (95%CIs) of hypertension according to different concordant/discordant groups across LDL-C 118 mg/dL and RC 20 mg/dL cut-points in the NHANES 1999 to 2018 excluding those taking prescription for hypertension (n=682)

|  | Concordant/discordant of LDL-C and RC, OR (95%CI) | | | |
| --- | --- | --- | --- | --- |
|  | **Low LDL-C and low RC** | **Low LDL-C and high RC** | **High LDL-C and low RC** | **High LDL-C and high RC** |
| Model 1^*^ | Ref | 2.06 (1.73-2.44) | 1.04 (0.86-1.26) | 1.79 (1.55-2.07) |
| Model 2^†^ | Ref | 1.97 (1.66-2.34) | 1.07 (0.88-1.30) | 1.76 (1.52-2.04) |
| Model 3^‡^ | Ref | 1.95 (1.64-2.31) | 1.07 (0.88-1.31) | 1.75 (1.50-2.04) |
| Abbreviations: OR, odds ratio; CI, confidence interval; LDL-C, low-density lipoprotein cholesterol; RC, remnant cholesterol.  ^*^ Model 1: adjusted for age + sex + race/ethnicity + educational level + family income-poverty ratio + smoking status + alcohol drinking.  ^†^ Model 2: adjusted for age + sex + race/ethnicity­­­ + educational level + family income-poverty ratio + smoking status + alcohol drinking + chronic kidney disease + coronary heart disease + diabetes mellitus.  ^‡^ Model 3: adjusted for age + sex + race/ethnicity + educational level + family income-poverty ratio + smoking status + alcohol drinking + chronic kidney disease + coronary heart disease + diabetes mellitus + eGFR + FBG + HbA1c.  All estimates accounted for complex survey design. | | | | |

***Table S18*** Adjusted ORs (95%CIs) of hypertension according to different concordant/discordant groups across LDL-C 118 mg/dL and RC 20 mg/dL cut-points in the NHANES 1999 to 2018 using different equations to recalculate LDL-C and RC

|  | Concordant/discordant of LDL-C and RC, OR (95%CI) | | | |
| --- | --- | --- | --- | --- |
|  | **Low LDL-C and low RC** | **Low LDL-C and high RC** | **High LDL-C and low RC** | **High LDL-C and high RC** |
| **Friedewald equation** | | | | |
| Model 1^*^ | Ref | 2.14 (1.82-2.52) | 1.06 (0.88-1.26) | 1.84 (1.58-2.14) |
| Model 2^†^ | Ref | 2.04 (1.73-2.40) | 1.09 (0.91-1.31) | 1.82 (1.55-2.12) |
| Model 3^‡^ | Ref | 2.00 (1.69-2.36) | 1.09 (0.91-1.31) | 1.79 (1.53-2.10) |
| **Sampson-NIH formula** | | | | |
| Model 1^*^ | Ref | 2.10 (1.78-2.48) | 1.03 (0.88-1.22) | 1.88 (1.62-2.19) |
| Model 2^†^ | Ref | 1.99 (1.69-2.36) | 1.06 (0.90-1.26) | 1.86 (1.60-2.17) |
| Model 3^‡^ | Ref | 1.95 (1.65-2.31) | 1.06 (0.90-1.26) | 1.84 (1.57-2.15) |
| Abbreviations: OR, odds ratio; CI, confidence interval; LDL-C, low-density lipoprotein cholesterol; RC, remnant cholesterol.  ^*^ Model 1: adjusted for age + sex + race/ethnicity + educational level + family income-poverty ratio + smoking status + alcohol drinking.  ^†^ Model 2: adjusted for age + sex + race/ethnicity + educational level + family income-poverty ratio + smoking status + alcohol drinking + chronic kidney disease + coronary heart disease + diabetes mellitus.  ^‡^ Model 3: adjusted for age + sex + race/ethnicity + educational level + family income-poverty ratio + smoking status + alcohol drinking + chronic kidney disease + coronary heart disease + diabetes mellitus + eGFR + FBG + HbA1c.  All estimates accounted for complex survey design. | | | | |
