# Supplementary figures and images for: Remnant cholesterol associates with hypertension beyond low-density lipoprotein cholesterol among the general US adult population
Source: Front Endocrinol (Lausanne). 2023 Sep 29;14:1260764. doi: 10.3389/fendo.2023.1260764 (PMC10570462; doi:10.3389/fendo.2023.1260764)

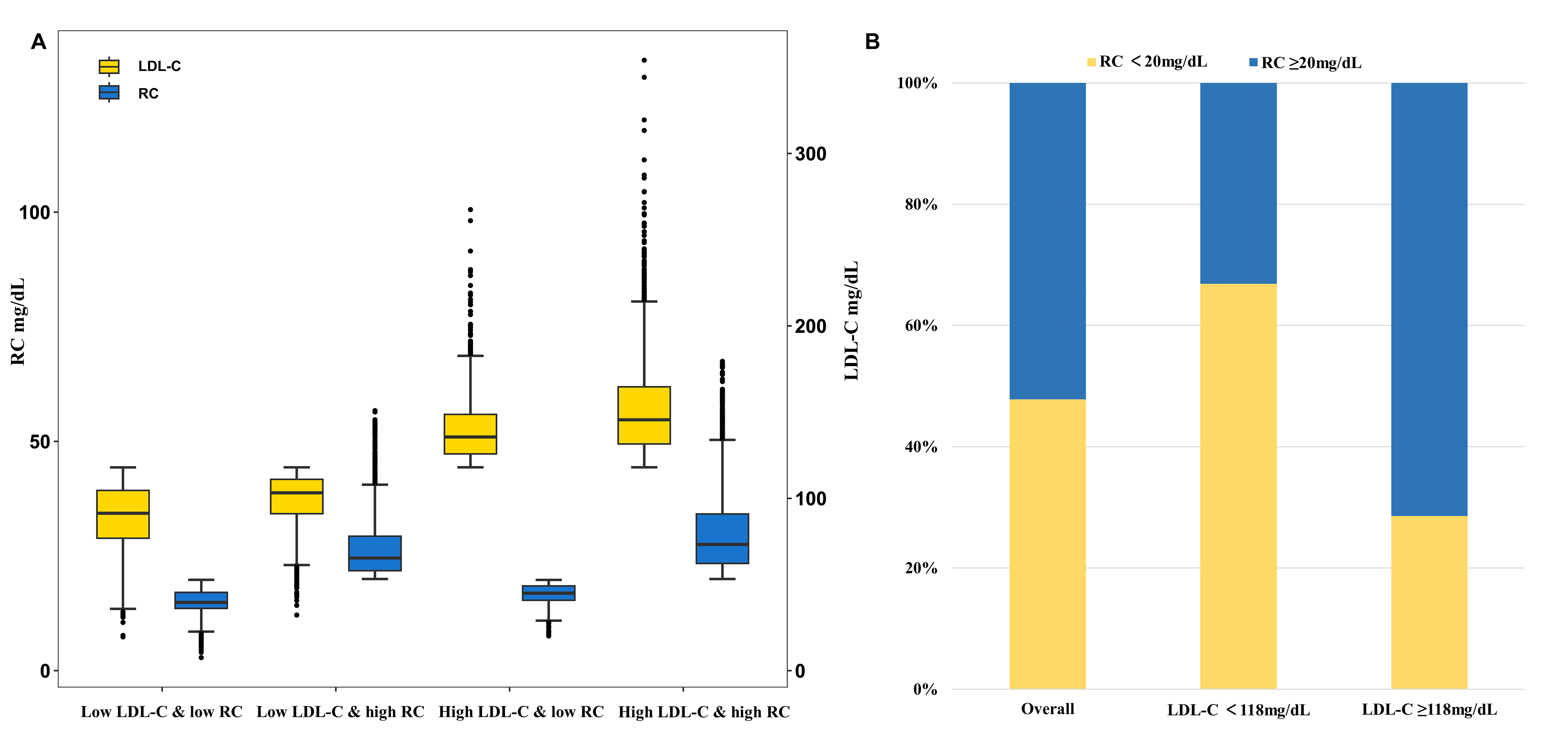

Supplement: Supplementary Figure 1 — The distribution of LDL-C and RC by the concordant/discordant groups across LDL-C 118 mg/dL and RC 20 mg/dL cut-points. (A) The box plot of LDL-C and RC concentrations for the different concordant/discordant groups. (B) Proportions of LDL-C and RC concordance and discordance. Percentages were weighted. LDL-C, low-density lipoprotein cholesterol; RC, remnant cholesterol. [file Image_1.tif]
